# Spatial Distribution of Forensically Significant Blow Flies in Subfamily Luciliinae (Diptera: Calliphoridae), Chiang Mai Province, Northern Thailand: Observations and Modeling Using GIS

**DOI:** 10.3390/insects9040181

**Published:** 2018-12-03

**Authors:** Tunwadee Klong-klaew, Ratchadawan Ngoen-klan, Kittikhun Moophayak, Kom Sukontason, Kim N. Irvine, Jeffery K. Tomberlin, Hiromu Kurahashi, Theeraphap Chareonviriyaphap, Pradya Somboon, Kabkaew L. Sukontason

**Affiliations:** 1 Department of Parasitology, Faculty of Medicine, Chiang Mai University, Chiang Mai 50200, Thailand; somtunwa@gmail.com (T.K.-k.); kom.s@cmu.ac.th (K.S.); pradya.somboon@cmu.ac.th (P.S.); 2 Department of Entomology, Faculty of Agriculture, Kasetsart University, Bangkok 10900, Thailand; ngernklun@yahoo.com (R.N.-k.); faasthc@ku.ac.th (T.C.); 3 Mahidol University, Nakhonsawan Campus, Nakhonsawan 60130, Thailand; khun_khithop@hotmail.com; 4 National Institute of Education, Nanyang Technological University, 50 Nanyang Avenue, Singapore 639798, Singapore; kim.irvine@nie.edu.sg; 5 Department of Entomology, Texas A&M University, 2475 TAMU, College Station, TX 77843, USA; jktomberlin@tamu.edu; 6 Department of Medical Entomology, National Institute of Infectious Diseases, Tokyo 162-8640, Japan; MLB15110@nifty.com

**Keywords:** *Lucilia*, *Hemipyrellia*, prediction, spatial distribution, Thailand

## Abstract

Blow flies of the subfamily Luciliinae (Diptera: Calliphoridae) are one of the main forensically important subfamilies globally. In addition to being used to estimate the minimum post-mortem interval (PMI_min_), assuming colonization occurred after death, blow fly specimens found infesting a human corpse are used to determine if the corpse was relocated or if the individual ingested narcotics prior to death. The presence of these blow flies in a given area is strongly influenced by abiotic and biotic factors, such as temperature, elevation, and habitat. Having this information, along with geographical distributions and the characteristics of preferred habitats, is necessary to better understand the biology of this group. This study aimed to characterize the spatial distribution of Luciliinae throughout 18 sampling sites within six ecozones (disturbed mixed deciduous forest, mixed deciduous forest, mixed orchard, paddy field, lowland village, and city/town) in central Chiang Mai Province, northern Thailand over one year (May 2009–May 2010). The purpose of the study was to elucidate the relationship of blow fly species composition with environmental abiotic factors (e.g., temperature, relative humidity, light intensity), and to predict the distribution of the common species within this subfamily using GIS. Adult collections were performed biweekly, baited with one-day-old beef offal. A total of 2331 Luciliinae flies trapped, comprising eight species, of which the four predominant species were *Hemipyrellia ligurriens* (Wiedemann) (*n* = 1428; 61.3%), *Lucilia porphyrina* (Walker) (*n* = 381; 16.3%), *Hemipyrellia pulchra* (Wiedemann) (*n* = 293; 12.6%), and *Lucilia papuensis* Macquart (*n* = 129; 5.5%). Population density across species varied seasonally, peaking in August 2009 coinciding with the rainy season. Predicting population composition was based on a model developed with ArcGIS 9.2, which utilized environmental variables (temperature, relative humidity, and light intensity) in conjunction with abundance data. Models indicated *H. ligurriens* had the most widespread geographic distribution, while *H. pulchra* was predicted to occur largely in mixed orchards and lowland villages. *Lucilia porphyrina* and *L. papuensis* were less widespread, restricted mainly to mixed deciduous forest. This model, along with knowledge of forensic information, may be useful under certain investigations where the corpse may have been relocated.

## 1. Introduction

Blow flies (Diptera: Calliphoridae) draw much attention from the forensic community due to their close association with decomposing remains and subsequent value as evidence [1,2,3,4]. They are the first group of insects to arrive at a corpse, often within minutes of death, thus being used in the crime scene investigation especially in estimating a minimum postmortem interval (PMI_min_) [3,5,6]. In many countries, blow flies of the subfamily Chrysomyinae (e.g., genus *Chrysomya*) account for the predominant taxa found infesting human corpses; however, those of the subfamily Luciliinae (e.g., genus *Lucilia*, *Hemipyrellia*, *Hypopygiopsis*) are still of consequence because of their distribution and close association with such resources [7,8,9,10]. Examples of cases where remains were infested with Luciliinae flies included *L. papuensis* in Australia [11], *H. ligurriens* in Malaysia [12], China [8], and Australia [11], *Hemipyrellia tagaliana* (Bigot) in Malaysia [13], and *Hypopygiopsis violacea* Macquart in Malaysia [14]. In Thailand, 10 species of Luciliinae flies have been recorded, including the genera *Lucilia*, *Hemipyrellia*, and *Hypopygiopsis* [15]. Among these, *H. ligurriens*, *Lucilia cuprina* (Wiedemann), and *L. porphyrina*, were found in association with human corpses [4,16]. Although the Luciliinae flies found in Thailand occur mainly in Asia, Australia, and Oceania [17,18,19,20,21], very little information about their spatial and temporal distributions and forensic cases have been reported. This limitation may be due to Luciliinae flies accounting for a small proportion of the populations that infest dead bodies. A recent study in Thailand reported that Luciliinae flies accounted for only 0.63% of a total of 147,248 calliphorids collected throughout a year [22]. Furthermore, Luciliinae accounted for 5.9% of blow flies collected from human corpses in Malaysia [12] and 6.5% of blow flies from cases in Thailand [4]. 

Knowledge of the distribution, biology, and behavior of forensically important flies is helpful in forensic investigations by providing information about time, location, and condition of the death [2,3,23,24]. In Thailand, the majority of studies on Luciliinae have focused on species identification [25,26,27] and developmental rate of the immature stages [28,29]. Although surveys of forensically important flies have been conducted on a local scale in Thailand, a comprehensive landscape assessment of the distribution of Luciliinae that are forensically important is limited [30,31,32]. Previous work on spatial analysis of forensically important blow flies has focused on *Chrysomya megacephala* (Fabricius) [33], *Chrysomya rufifacies* (Macquart) [34], *Chrysomya pinguis* (Walker)*, Chrysomya chani* Kurahashi, *Chrysomya villeneuvi* Patton and *Ceylonomyia nigripes* (Aubertin) [35]. However, studies on Luciliinae flies are lacking. Thus, this study aimed to investigate the occurrence of Luciliinae blow flies across six diverse land use categories in central Chiang Mai Province, northern Thailand. The influence of climatic factors (temperature, relative humidity, and light intensity) on their geographic distributions was investigated. Furthermore, the predicted distributions of the predominant species sampled were modeled using ArcGIS 9.2 (ESRI, Redlands, CA, USA). To our knowledge, this is the first study to spatially and temporally characterize the Luciliinae fly population over a variety of land use types in Thailand.

## 2. Materials and Methods

### 2.1. Study Areas

**Figure 1:**
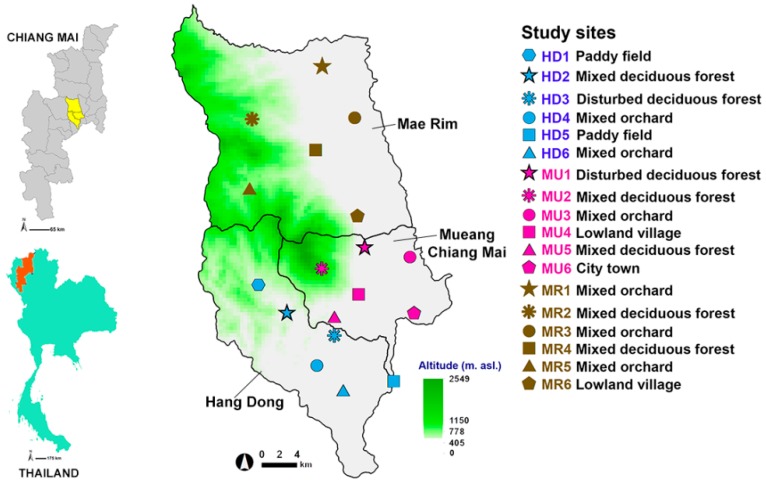
Map of Thailand showing three sample districts (Mueang Chiang Mai, MU; Mae Rim, MR; and Hang Dong, HD) and 18 sample locations. Green shading indicates a mountainous zone.

In order to develop a prediction model, flies of forensic importance were sampled in May 2009 to May 2010 by selecting 18 study sites located within three districts of Chiang Mai Province, northern Thailand. These calibrated study sites were distributed across the following districts: one urban (Mueang Chiang Mai (MU)) and two suburban sites (Hang Dong (HD) and Mae Rim (MR)) (Figure 1). Following a systematic random sampling method [36], the study area within the three districts was partitioned into a sampling frame of 5 × 5 km for suburban areas (Mae Rim and Hang Dong districts) and 3 × 3 km for the urban area (Mueang Chiang Mai district). The study area was plotted using the contour maps of Chiang Mai Province (MapMagic™ scale 1:150,000 with a UTM projection type, Everest Spheroid and the Indian 1975 Datum). More detail of the sample site selection procedure and land use classification is provided in File S1. Six land uses were categorized: mixed deciduous forest, disturbed mixed deciduous forest, paddy field, mixed orchard, lowland village, and city/town. The 18 study sites covering six land uses were used to model the likely spatial distribution of Luciliinae flies. 

### 2.2. Adult Flies Collection

Adult flies were trapped every two weeks from May 2009 to May 2010, using an in-house prototype portable funnel trap kit [33]. The trapping method was described previously [33]. Briefly, the trap consisted of a polyvinyl chloride (PVC) frame box (30 × 30 × 50 cm), a fly entrance module, and a black fly net (30 × 30 × 80 cm). Two replicate sets of traps were placed in a single row (50 m apart) on the ground [33], each baited with 250 g of one-day-old beef offal [37], which was kept in a translucent plastic container. The bait was placed underneath the fly entrance module after it had been tied to a fly net with elastic bands and installed with the PVC frame box. Flies lured by the smell from the offal were collected by their passive movement up toward the light through the fly entrance module to the fly collection net after landing on the bait. Traps were placed in the shade to prevent thermal stress for trapped flies [38]. The collection was done for a 1-h period between 09:30 a.m. and 12:00 p.m. Physical data of each study site were collected including light intensity (lux) (LUX/FC light meter TM-204 Tenmars, Tenmars Electronics Co., Ltd., Taipei, Taiwan), temperature (°C), relative humidity, RH (%) (Digital Hygro-Thermo Meter (DHT-1), Daeyoon Scale Industrial Co., Ltd., Seoul, South Korea) and the co-ordinates (Garmin™ eTrex Handheld GPS, Garmin China Co., Ltd., Chaoyang, China).

All specimens were carried to the laboratory of the Department of Parasitology, Faculty of Medicine, Chiang Mai University. They were sacrificed by placement in a freezer set at 0 °C for 2 h. Flies were then identified under a dissecting microscope (model SZ2-ILST, Olympus Corporation, Tokyo, Japan) using taxonomic keys, sexed, and counted [20,21]. 

### 2.3. Statistical Analysis

Three seasons were considered: a rainy season occurring from June through October, winter from November through February, and summer from March through May (File S2). Species sampled with fewer than 100 specimens captured year-round were excluded from the analysis. We analyzed the relationship of environmental variables (temperature, relative humidity, and light intensity) with trap catch using negative binomial regression analysis. We used one-way analysis of variance (ANOVA) followed by the post hoc Bonferroni test (homogeneity of variance: *p* > 0.05) or Dunnett T3 test (homogeneity of variance: *p* < 0.05) to determine the relationship between land use types and mean total number of flies using IBM SPSS Statistics for Windows, version 22.0 (IBM Corp., Armonk, NY, USA) (α = 0.05). 

To predict the spatial distribution of Luciliinae flies by season and aggregated for the full year, we conducted additional geospatial analysis using a kriging/co-kriging approach in ArcGIS 9.2. Kriging is a kind of linear least squares estimation method [39] that is associated with spatial optimal linear prediction in which the unknown random-process mean is estimated with the best linear unbiased estimator [40]. To create a continuous surface of the phenomenon, predictions are made for each location in the study area based on the semivariogram and the spatial arrangement of measured values that are nearby. Co-kriging is an extension of kriging for prediction of one variable using other variables. The co-variables must have a relationship and this relationship must be defined [39]. The use of co-kriging requires the spatial covariance model of each variable and the cross-covariance model of the variables is defined [39].

In this study, the parameters that were used in co-kriging included climatic factors that had a statistically significant relationship with fly numbers and the total trap catch during the study period. The data were log-transformed to normalize the distribution and minimize standard error of the geographical analysis [40]. Moreover, the data of land use types were defined as dummy variables and were incorporated into the ordinary kriging/co-kriging analysis. The mathematical models for evaluating the semivariogram/covariance function were accepted when the model provided the lowest root-mean-square prediction error [33].

## 3. Results

**Figure 2:**
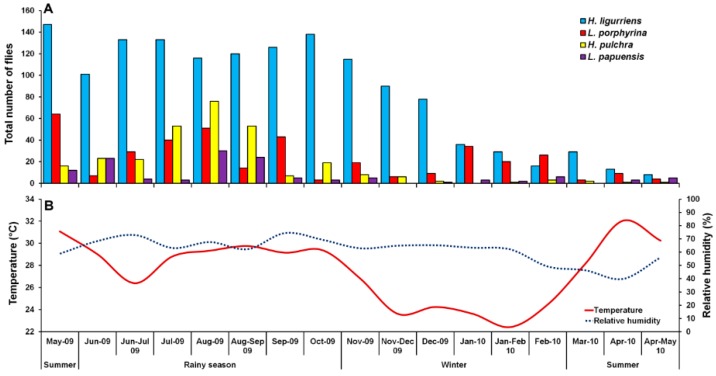
Monthly fluctuations in trap catches of *H. ligurriens*, *L. porphyrina*, *H. pulchra*, and *L. papuensis* determined using a portable funnel trap baited with one-day-old beef offal in Chiang Mai Province, northern Thailand, May 2009 to May 2010 (**A**), and variation of temperature and relative humidity recorded during the fly survey (**B**).

A total of 2331 Luciliinae flies were sampled, comprising eight species, of which the predominant species was *H. ligurriens*, representing 61.3% of the individuals (*n* = 1428). Other associated species were *L. porphyrina* (*n* = 381; 16.3%), *H. pulchra* (*n* = 293; 12.6%), and *L. papuensis* (*n* = 129; 5.5%). Small numbers of *L. cuprina* (*n* = 67; 2.9%), *Hypopygiopsis tumrasvini* Kurahashi (*n* = 23; 1.0%), *Lucilia sinensis* Aubertin (*n* = 6; 0.3%) and *Hypopygiopsis infumata* (Bigot) (*n* = 4; 0.2%) (Table 1). Therefore, this paper focused mainly on the four most sampled species, namely *H. ligurriens*, *L. porphyrina*, *H. pulchra*, and *L. papuensis*. The sampled fly numbers varied seasonally, peaking in August 2009, coinciding with the rainy season, while a minor peak was in summer (May 2009). The sample numbers decreased gradually during the late rainy season (September 2009) and remained low throughout the winter and summer (Figure 2). 

**Figure 3:**
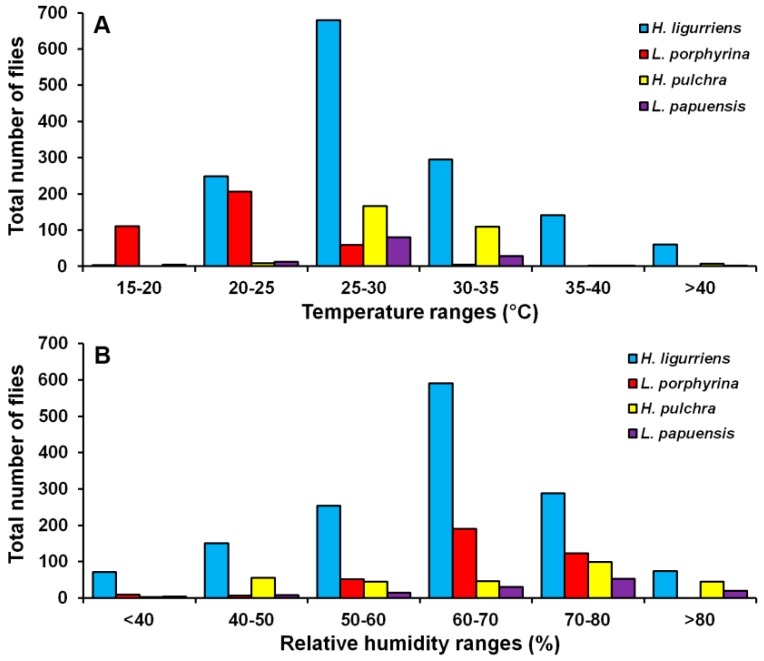
Total number of flies captured at different temperature (**A**) and relative humidity ranges (**B**).

**Figure 4:**
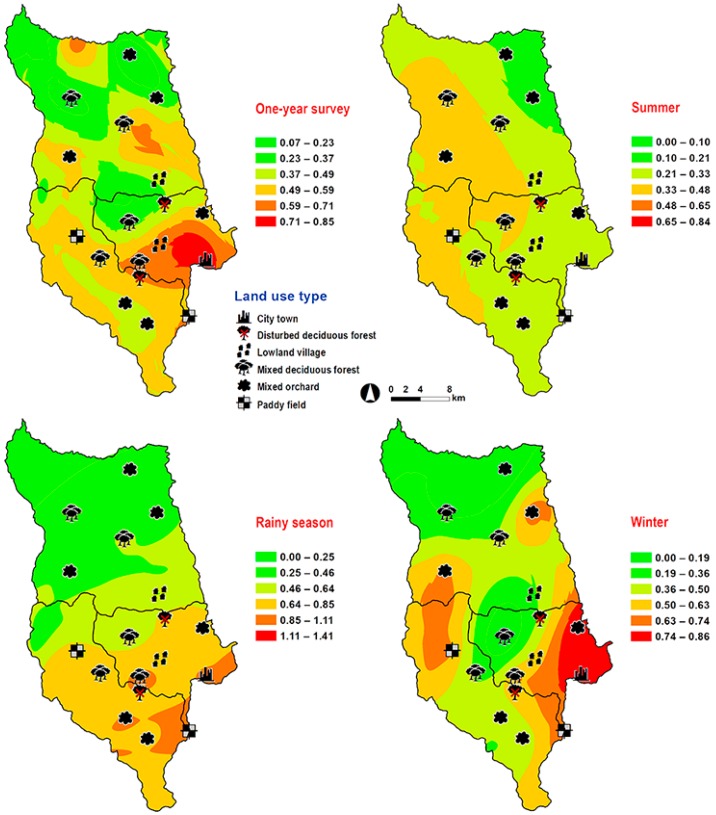
Predictive distribution maps of *Hemipyrellia ligurriens*. The color scheme reflects different fly density categories. The red areas indicate the highest fly population, while the green areas indicate the lowest population density. The scale corresponds to natural logarithm of (fly density + 1).

*Hemipyrellia ligurriens* had widespread geographic distribution (Table 1). Although this species was sampled throughout the year, a bimodal peak was observed—the major peak occurring in late summer (May 2009), while a minor peak occurred in the rainy season (June–October 2009). As expected, sample numbers gradually decreased at the onset of winter (November 2009) and remained minimal throughout winter and summer (January–May 2010) (Figure 2). *H. ligurriens* trap catch was positively correlated with temperature and relative humidity (*p* < 0.05), with increased temperature (*B* = 0.103, *p* = 0.0001) and relative humidity (*B* = 0.035, *p* = 0.0001) associated with increases in trap catch (Table 2). No correlation with light intensity was found (*p* = 0.954). The greatest numbers were collected at 25–30 °C and 60–70% RH (Figure 3). Analyses using co-kriging showed the spatial distribution of fly density across a variety of land uses in Mueang Chiang Mai district (Figure 4).

**Figure 5:**
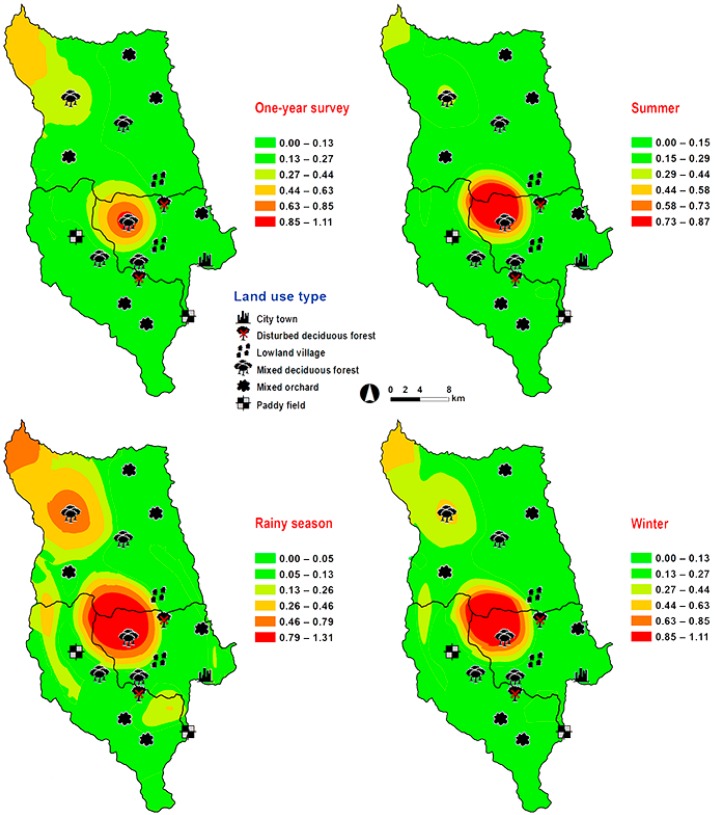
Predictive distribution maps of *Lucilia porphyrina.* The color scheme represented different fly density categories. The red areas represent the highest fly population, while the green represent the lowest population density. The scale corresponds to natural logarithm of (fly density + 1).

*Lucilia porphyrina* was prevalent in mixed deciduous forest (Table 1). Although it was trapped throughout the year, no particular seasonal fluctuation pattern was found. Collections were highest in late summer (May 2009), and in the rainy season (June–September 2009), while a small peak was in the winter (January–February 2010) (Figure 2). *L. porphyrina* trap catch was positively correlated with temperature and light intensity (*p* < 0.05), with increased temperature (*B* = −0.263, *p* = 0.0001) and light intensity (*B* = −0.000034, *p* = 0.0001) significantly associated with the decreased trap catch. No correlation with relative humidity was found (*p* = 0.741) (Table 2). The greatest numbers of this species was collected at 20–25 °C and 60–70% RH (Figure 3). Analyzed using co-kriging, the predicted geographical distributions were found in mixed deciduous forest at 950 m a.s.l. of Mueang Chiang Mai district (MU2) and mixed deciduous forest at 407 m a.s.l. of Mae Rim district (MR2) throughout the year (Figure 5).

**Figure 6:**
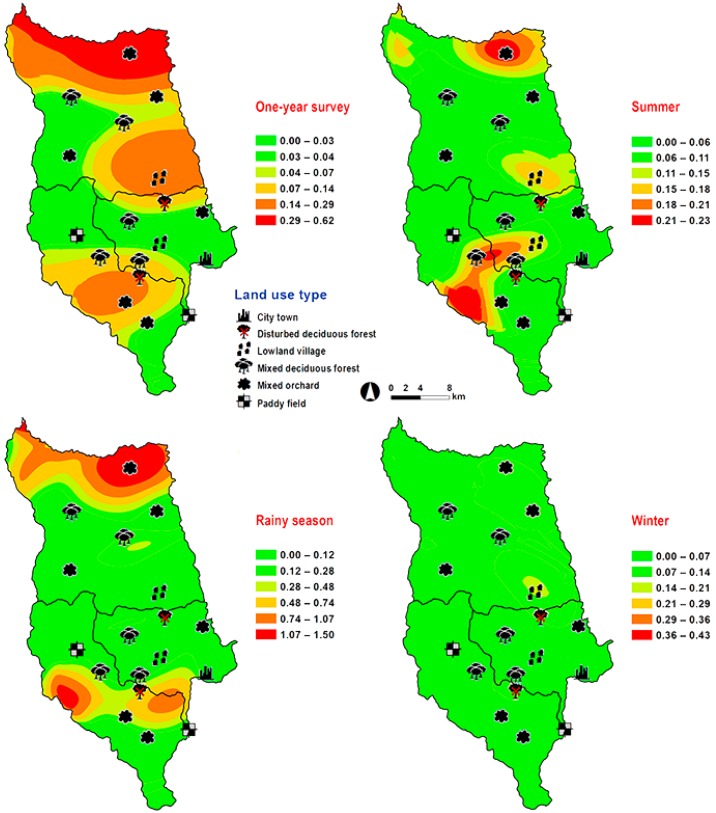
Predictive distribution maps of *Hemipyrellia pulchra.* The color scheme represented different fly density categories. The red areas represent the highest fly population, while the green represent the lowest population density. The scale corresponds to natural logarithm of (fly density + 1).

*Hemipyrellia pulchra* was collected primarily in mixed orchard and lowland village; this species was not found in paddy field or city/town (Table 1). Peak population was observed in the rainy season (August 2009), and decreased gradually in late rainy season (September 2009) throughout winter (November 2009–February 2010) and summer (March–May 2010). Regression analysis showed a positive significant association between trap catch of *H. pulchra* and climatic factors (temperature and relative humidity) (*p* < 0.05). Increased temperature (*B* = 0.325, *p* = 0.0001) and relative humidity (*B* = 0.077, *p* = 0.0001) were significantly associated with increased trap catch. No correlation with light intensity was found (*p* = 0.0578) (Table 2). The greatest number of this species was captured at 25–30 °C and 60–70% RH (Figure 3). Co-kriged prediction maps were produced, showing abundance in mixed orchard and lowland village. In summer, the spatial pattern was similar to the aggregated one-year survey. This species was predicted to occur largely in mixed orchard and disturbed mixed deciduous forest in the rainy season, while the fewest numbers were predicted in winter, with little apparent spatial variation (Figure 6).

**Figure 7:**
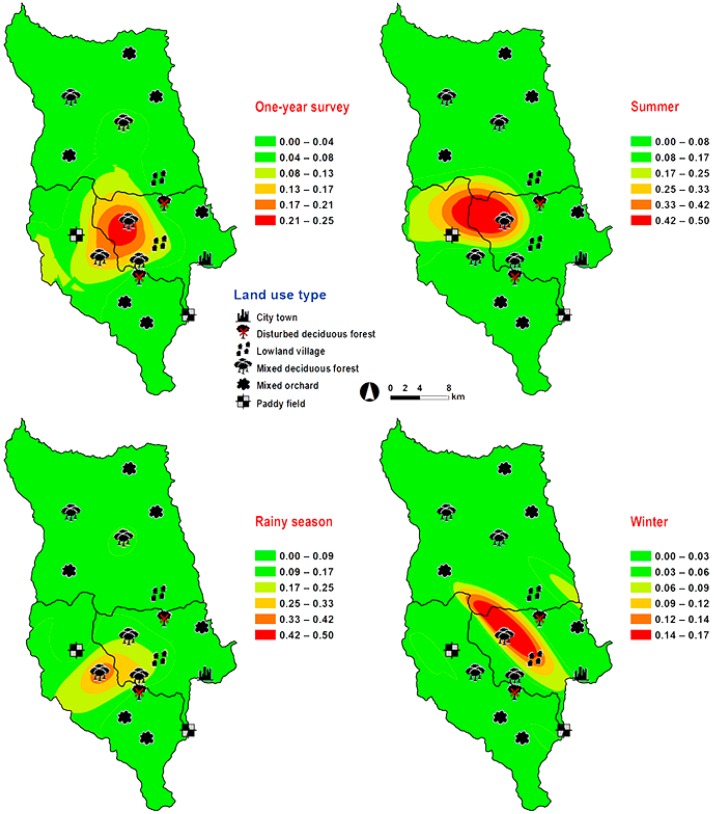
Predictive distribution maps of *Lucilia papuensis.* The color scheme represented different fly density categories. The red areas represent the highest fly population, while the green represent the lowest population density. The scale corresponds to natural logarithm of (fly density + 1).

*Lucilia papuensis* was significantly prevalent (ANOVA, *p <* 0.05) in mixed deciduous forest and lowland village, respectively (Table 1). A bimodal peak population was evident, with a major peak in the rainy season (August–September 2009) and a minor peak at the onset of the rainy season (June 2009). Fly numbers decreased dramatically during the late rainy season (September–October 2009) throughout winter and summer. No flies of this species were sampled in November‒December 2009 and March 2010 (Figure 2). The trap catch of this species was significantly associated with the climatic factors (*p* < 0.05). Increased temperature (*B* = 0.126, *p* = 0.022) and relative humidity (*B =* 0.033, *p* = 0.048) were significantly associated with increased trap catch. However, the increase of light intensity was associated with a significantly reduced trap catch of flies (*B* = −0.00003, *p* = 0.003) (Table 2). The greatest numbers of flies were collected at 25–30 °C and 70–80% RH (Figure 3). Co-kriged prediction maps were produced. A one-year survey and seasonal prediction maps exhibited a similar pattern, as this species was predicted to occur largely in mixed deciduous forest along the mountainous areas and in lowland villages (Figure 7). 

## 4. Discussion

An adequate knowledge of the ecology and geographical abundance of blow flies is important for the forensic sciences [2,3,41]. However, in Thailand, such information on geographic distribution of forensically important flies was limited prior to this study, which is the first to report the distribution of four forensically important Luciliinae flies; *H. ligurriens*, *H. pulchra*, *L. porphyrina*, and *L. papuensis*, over a full year. Maps of predicted fly distribution were generated using the Geostatistical Analyst Extension of ArcGIS 9.2.

Abundance relative to ecotype differed among the four species. The study sites, which represented different broad ecological locations, were chosen based on a systematic random sampling method. Some of the 18 study sites under the six land use categories, covering the city to forested areas, may not be suitable for some fly species [17,20,21,42]. We found *H. ligurriens* had widespread geographic distribution, while *H. pulchra* was collected mainly in mixed orchard and lowland village land uses. This result may reflect that *H. ligurriens* can live and develop in many substances such as animal dung, carcasses, human feces, and decomposed organic matter [17,19], while the adult of *H. pulchra* prefer flowers and fruits in the orchards [19]. A study in Phitsanulok Province, northern Thailand, also reported a wide variety of habitat types, where *H. ligurriens* can be found, ranging from residential areas to the mountainous zones, but *H. pulchra* was collected only in the agricultural areas and in the forests [43]. A previous study reported various altitude distribution ranges of *H. ligurriens*, indicating greater adaptation compared with *H. pulchra* [32]. 

*Lucilia porphyrina* and *L. papuensis* were less widespread, and were restricted mainly to the mixed deciduous forest (at 950 m a.s.l.). Likewise, others have reported that *L. porphyrina* and *L. papuensis* are likely to have their distribution restricted to forested highland areas in Thailand [21,32,43]. A study in Japan indicated that the females of *L. porphyrina* preferred to lay their eggs in the mountains and/or highlands [44]. Additionally, their larvae have been reported to feed on animals’ carcasses [19,21], which could be found in the forests. However, *L. porphyrina* could be found in human residences in India and in the mainland of Japan (for overwintering) [19,44]. A small number of *L. papuensis* was collected in the present study, but it was found in all six of the land uses, with the greatest number in mixed deciduous forest. Many studies also supported its preference for the forest areas [17,19,42,43]. This may be caused by the adults gathering around decaying animals (e.g., earthworms, land snails, and snakes) and other vertebrate carcasses [19,21] that could be found in the forest areas. 

The peak trap catch of these species was found in the rainy season (August 2009), and the nadir occurred during the summer (March–May 2010). This finding is in contrast with many studies where more blow flies were collected during the warmer months [22,45,46]. The conditions in the rainy season (mean temperature 29.1 ± 0.3 °C, mean RH 69.7 ± 1.0%) may be suitable for the development of Luciliinae flies. A previous study with *H. ligurriens* suggested the optimal condition for the development of this species is a temperature range of 16 to 28 °C, not 31 °C or above [47]; therefore, the temperature of the summer months in this study (mean temperature 30.6 ± 0.5 °C) (see Figure 2) may exceed the optimal range. However, the present study was carried out during a single year. To obtain reliable seasonal patterns, the season needs to be replicated (i.e., the study should be replicated during more than one year). In this study, fly trapping was done before noon (9:30 a.m. and 12:00 p.m.). Blow fly activity was reported to be higher around noontime and in the afternoon than in the early morning [22,48,49], so our collection time may have affected the resulting collections. 

The yearly sampling across 18 study sites allows us to spatially predict the population distribution of Luciliinae flies. Our study is not the first to model the prediction of forensically important blow flies. Previous investigations have shown the spatial models of *C. megacephala*, *C. rufifacies*, *C. pinguis*, *C. chani*, *C. villeneuvi* and *Cey. nigripes* [33,34,35]. According to this study, *H. ligurriens* was predicted to occur across a variety of land uses in Mueang Chiang Mai district especially nearby residential areas (e.g., city/town and lowland village), which is similar to *C. megacephala* and *C. rufifacies* [33,34]. On the other hand, *L. porphyrina* and *L. papuensis* showed preference in the mixed deciduous forest, which is consistent with *C. pinguis*, *C. chani*, *C. villeneuvi* and *Cey. nigripes* [35]. The greatest number of *H. pulchra* was predicted mainly in mixed orchard and lowland village, with low altitudes ranging between 300 and 400 m a.s.l., which is in contrast with a previous study that found this species only at 901–1050 m a.s.l. [32]. However, their studies were carried out in the forested area of altitudes ranging from 400–1050 m a.s.l. and only one specimen of *H. pulchra* was collected by a sweep net.

According to our results, the prediction maps indicate *H. ligurriens* occurred in a variety of habitats and is closely associated with human activity. Previous studies reported *H. ligurriens* infested a human corpse in a forested area of northern Thailand [4]. This species has also been associated with human remains in an outdoor environment of Australia [11]. The widespread distribution of *H. ligurriens* (ranging from residential areas, forests, and highland) was also observed in Bangladesh and Malaysia [7,42]. In contrast, *L. porphyrina* was predicted mainly in the mixed deciduous forest at 905 m a.s.l. This species may be used as insect evidence when corpses are found in the forest of mountainous areas. Recently, *L. porphyrina* was found on a human corpse discovered in an open mountainous area (1200 m a.s.l.) during the winter time of northern Thailand [16]. Additionally, this species infested rabbit carcasses in the highlands of Malaysia (1517.3 m a.s.l.), suggesting that it is a forensically important species in the highlands.

## 5. Conclusions

We provide predictive maps of the spatial distribution for forensically important blow flies within the subfamily Luciliinae as related to land use types and climatic factors. *Hemipyrellia ligurriens* and *H. pulchra* have spread through a wide range of land use types, implying that their application for explaining the location of corpse movement is limited, especially when the immature specimens of these species were found. On the other hand, *L. porphyrina* and *L. papuensis* evidently show a preference to the mixed deciduous forest. This database may be incorporated as a useful tool in forensic entomology. To be used for PMI_min_ estimation, biological information such as developmental rate or insect succession of these species are mandatory.

## Figures and Tables

**Table 1:** Climatic factors (temperature, relative humidity, and light intensity) recorded and total numbers of Luciliinae flies collected at each land use type.

	Land Use Types					
	Mixed Deciduous Forest	Disturbed Mixed Deciduous Forest	Mixed Orchard	Paddy Field	Lowland Village	City/Town
Climatic factor recorded *						
Temperature (°C)	26.5 (16.7–39.8)	28.4 (21.0–39.3)	28.4 (19.7–45.2)	27.5 (19.6–40.8)	28.3 (18.7–35.8)	25.8 (18.8–30.7)
Relative humidity (%)	67.0 (35–87)	61.5 (35–80)	59.0 (25–89)	63.5 (39–83)	65.0 (36–83)	64.0 (40–78)
Light intensity (Lux)	26,550 (193–107,500)	59,403 (2320–359,600)	47,200 (1000–118,500)	36,300 (6700–112,000)	38,550 (2900–95,700)	22,000 (442–94,000)
Number of adult flies collected **						
*Hemipyrellia ligurriens* (Wiedemann)	4.70 ± 1.00 a	4.53 ± 1.13 a	4.16 ± 0.73 a	5.71 ± 0.95 a	4.32 ± 1.13 a	7.87 ± 2.18 a
*Hemipyrellia pulchra* (Wiedemann)	0.18 ± 0.06 ab	0.62 ± 0.22 a	2.25 ± 0.92 a	0 b	0.88 ± 0.29 a	0 b
*Lucilia porphyrina* (Walker)	4.43 ± 1.23 a	0 b	0.09 ± 0.05 b	0 b	0 b	0 b
*Lucilia papuensis* Macquart	1.21 ± 0.38 a	0.012 ± 0.06 b	0.05 ± 0.03 b	0.09 ± 0.09 b	0.41 ± 0.15 ab	0.07 ± 0.07 b
*Lucilia cuprina* (Wiedemann)	0.07 ± 0.04	0.03 ± 0.03	0.32 ± 0.09	0.06 ± 0.06	0.06 ± 0.06	1.60 ± 0.64
*Hypopygiopsis tumrasvini* Kurahashi	0.23 ± 0.11	0	0.02 ± 0.02	0.03 ± 0.03	0.03 ± 0.03	0
*Lucilia sinensis* Aubertin	0.05 ± 0.03	0	0	0	0.06 ± 0.06	0
*Hypopygiopsis infumata* (Bigot)	0.02 ± 0.02	0	0	0	0.06 ± 0.06	0

* Data presented as Median (min-max); ** Data presented as Mean ± SE; Different letter (a, b) shown as a significant difference within groups (ANOVA: *p* < 0.05).

**Table 2:** The association of climatic factors with Luciliinae trap catches.

Species	Factors	Regression Coefficient (*B*)	SE	95% CI	*p* Value
*H. ligurriens*	Temperature	0.103	0.0225	0.059, 0.147	0.0001
	RH	0.035	0.0092	0.017, 0.054	0.0001
	Light intensity	−0.0000002	0.0000032	−0.000006, 0.000006	0.954
*H. pulchra*	Temperature	0.325	0.0655	0.197, 0.453	0.0001
	RH	0.077	0.0217	0.035, 0.120	0.0001
	Light intensity	−0.000006	0.00001	−0.000025, 0.000014	0.0578
*L. porphyrina*	Temperature	−0.263	0.0325	−0.326, −0.199	0.0001
	RH	−0.003	0.0102	−0.023, 0.017	0.741
	Light intensity	−0.000034	0.000005	−0.000044, −0.000025	0.0001
*L. papuensis*	Temperature	0.126	0.0107	0.001, 0.043	0.022
	RH	0.033	0.0036	0.001, 0.015	0.048
	Light intensity	−0.00003	0.00001	−0.00005, −0.00001	0.003

## Supplementary Materials

The following are available online at http://www.mdpi.com/2075-4450/9/4/181/s1, File S1: Stratified random sampling, File S2: General description of Thailand climate.
