# Supplementary material for: Spatial Distribution of Forensically Significant Blow Flies in Subfamily Luciliinae (Diptera: Calliphoridae), Chiang Mai Province, Northern Thailand: Observations and Modeling Using GIS
Source: Insects. 2018 Dec 3;9(4):181. doi: 10.3390/insects9040181 (PMC6315425; doi:10.3390/insects9040181)
Supplement: Supplementary file 1 [file insects-09-00181-s001.zip › Suppl/File S1 Stratified random sampling.docx]

1. Study Site Selection

The sampling was carried out in the central part of Chiang Mai Province, including Mueang Chiang Mai (MU), Mae Rim (MR), and Hang Dong (HD) districts. The study plots were chosen based on a systematic random sampling method. The total area of the Mueang Chiang Mai district was smaller than the total area of the Mae Rim and Hand Dong districts. Therefore, the study area was stratified using a sampling frame of 3 × 3 km in the Mueang Chiang Mai district (MU) and 5 × 5 km in the suburban area of Mae Rim and Hang Dong districts (MR and HD), based on a map of Chiang Mai Province (MapMagic™ scale 1: 150,000 on the UTM projection type, Everest Spheroid and the Indian 1975 Datum) [1].

From this procedure, the sampling frame (S_n_) consisted of all the points (x- and y-coordinates) located in the intended geographical region and divided into a set of equal plots. As such, S_n_ was determined as: S_n_ = (x_min_, x_max_) (y_min_, y_max_), where *n* was a fixed plot number. Accordingly, the collection sites were selected using systematic sampling. This approach was achieved by sampling every *k*^th^ item from the sampling frame (S_n_) after the first *k* item was selected at random from a random number [2], with each item in the population having an equal probability of selection. The value of *k* was calculated as:

| $k=\frac{\mathrm{TS}_{n}}{n}$ | (1) |
| --- | --- |

where TS_n_ was the total number of sampling frames and *n* the number of study sites of interest. According to these procedures, 18 study sites (six from each district) were chosen from different land use types for fly collection.

To obtain the characteristics of land use types in specific areas, the Extract tool in ArcGIS 9.2, known as Clip, was implemented. The Clip tool was used to extract portions of features from an input feature class that overlapped with a clip feature class. In this study, the land use map of Chiang Mai Province was represented as an input feature and the clip feature classes were the boundaries of the Mueang Chiang Mai, Mae Rim and Hang Dong districts. After creating the land use map of an individual district, the co-ordinates of the trapping sites were loaded into ArcGIS 9.2 (Figure 1–3). The georeferencing and coordinate system wizard were used to organize the map unit, projection spheroid, and datum zone. All features were set in the UTM projection type, Everest Spheroid, and the Indian 1975 Datum. Furthermore, locations of the study site were overlaid on the thematic map derived from Landsat 5 (path/row: 131/470: band 5, acquisition times: 10 April 2010), supported by the Geo-Informatics and Space Technology Development Agency (Public Organization) [1].

2. General Description of Study Sites in Mueang Chiang Mai District

Mueang Chiang Mai district is the capital district of Chiang Mai Province. It is a center for the commercial and service industry. The co-ordinates, land use types, and altitude of the collection sites in Mueang Chiang Mai district are shown in Table 1.

**Table 1.** The study sites in Mueang Chiang Mai district.

| **Code** | **UTM Coordinate** | | **Location** | **Sub-district** | **Land use type** | **Altitude (m a.s.l.)** |
| --- | --- | --- | --- | --- | --- | --- |
|  | **x** | **y** |  |  |  |  |
| MU1 | 495312 | 2081814 | Nhong Hor | Chang Phueak | Disturbed mixed deciduous forest (DDF) | 385 |
| MU2 | 490465 | 2079323 | Tham Lue Sri | Suthep | Mixed deciduous forest (MDF) | 950 |
| MU3 | 500430 | 2080649 | Tha Kra Dad | Fah Ham | Mixed orchard (MO) | 309 |
| MU4 | 4994617 | 2076438 | U Mong | Suthep | Lowland village (LV) | 349 |
| MU5 | 491862 | 2068567 | Doi Kham | Pa Chee | Mixed deciduous forest (MDF) | 356 |
| MU6 | 500893 | 2074324 | Nhong Hoi | Nhong Hoi | City town (CT) | 311 |


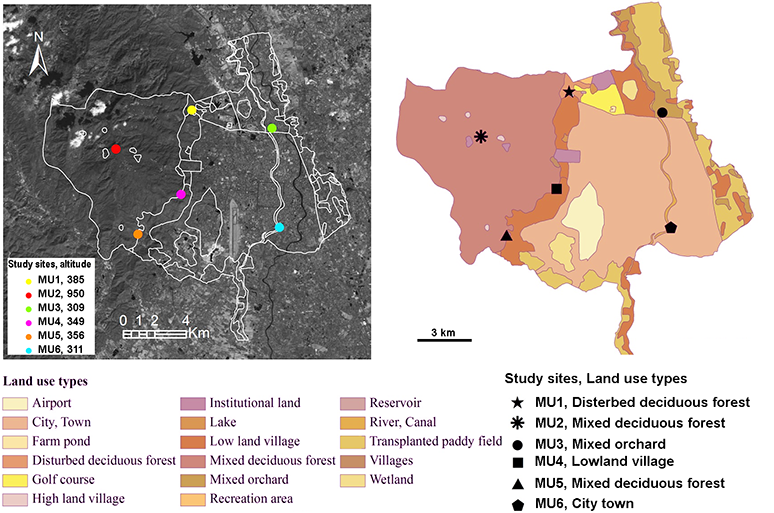


**Figure 1.** Maps representing the six study sites located in Mueang Chiang Mai district.

MU1 was defined as disturbed deciduous forest located on the foothills of Doi Suthep-Pui Mountain, which is a part of Doi Suthep-Pui National Park. The fly collections were done around a man-made reservoir inside a village. Garbage bins were found scattered around the study site.

MU2 was located in the mixed deciduous forest of Doi Suthep-Pui National Park at 950 m a.s.l. The study site was about 1 km from the community school. No households were found around the study area. Fly collection was carried out along a small natural stream. The climate conditions were stable, with cool and humid weather during the period of fly trapping. The light intensity was low because of a large canopy of trees.

MU3 was located on the west bank of the Ping River at an altitude of 309 m a.s.l. in the northeastern area of Mueang Chiang Mai district. A large human community, local market, and mixed orchard were found around this study site. The fly traps were placed in the area surrounded by the residential areas, longan orchards, and a frog farm.

MU4 was located in the center of Mueang Chiang Mai district at an altitude of 349 m a.s.l. The ecotype was classified as a lowland village. The fly traps were set in an abandoned area covered with bushes and trees located near the Choeng Doi Su Thep Wildlife Conservation Development and Extension Station.

MU5 was located in the southwestern area of Mueang Chiang Mai district at an altitude of 356 m a.s.l. The tourist attractions of the Royal Flora Ratchaphruek and Chiang Mai Night Safari were in the area of study. The fly traps were placed at the foothills of a mixed deciduous forest in the Suthep-Pui National Park. This study site was located nearby the Doi Kham Royal Project, which involves with the production of flowers and vegetables. Livestock (i.e., goats, chickens, and cows) was found at a distance of 1 km from this site.

MU6 was an urban study site with a large human community at an altitude of 311 m a.s.l. This area was defined as a city/town surrounded by commercial services, hotels, restaurants, households, and a fresh market. The fly traps were set around the National Housing Authority villages, about 1 km from the Nhong Hoi fresh market. Garbage bins were found around this site.

3. General Description of Study Sites in Mae Rim Ddistrict

Mae Rim district is a suburban area located to the north of Mueang Chiang Mai district. The co-ordinates, land use types and elevation of the study sites in Mae Rim district are given in Table 2.

**Table 2.** The study sites in Mae Rim district.

| **Code** | **Coordinate** | **Coordinate** | **Location** | **Sub-district** | **Land use type** | **Altitude**  **(m a.s.l.)** |
| --- | --- | --- | --- | --- | --- | --- |
|  | **x** | **y** |  |  |  |  |
| MR1 | 490497 | 2102275 | Saluang Nok | Saluang | Mixed orchard (MO) | 353 |
| MR2 | 482569 | 2096220 | Mae Ram | Mae Ram | Mixed deciduous forest (MDF) | 407 |
| MR3 | 494184 | 2096377 | Nhong Arb Chang | San Pong | Mixed orchard (MO) | 310 |
| MR4 | 489752 | 2092744 | Mae Ram | Mae Ram | Mixed deciduous forest (MDF) | 348 |
| MR5 | 482295 | 2088362 | Pong Yang Nai | Pong Yang | Mixed orchard (MO) | 746 |
| MR6 | 494507 | 2085306 | Huay Toeng Thao | Don Kaew | Lowland village (LV) | 335 |


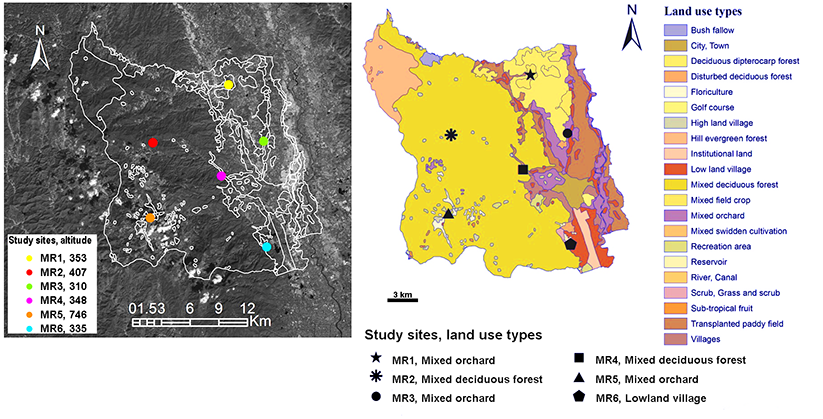


**Figure 2.** Maps representing the six study sites located in Mae Rim district.

MR1 was located in the northern area of Mae Rim district at an altitude of 353 m a.s.l. This study site was surrounded by mixed orchards such as longan, mango, and banana. Livestock (i.e., cows and pigs) was found at this site. The fly collection was conducted in an open area near a man-made reservoir.

MR2 was located in the northwestern area of Mae Rim district at an altitude of 407 m a.s.l. The fly traps were set along the roadside because this site was located on a steep hill of mixed deciduous forest. All traps were set under a bamboo shrub. No houses were found within a 1-km radius of this site, but a small cow herd was found sometimes.

MR3 was located at the low altitude of 310 m a.s.l. in the north-eastern area of Mae Rim district. It was a big agricultural area with a moderately sized human community located close to the main highway. The fly collection was conducted in a field of mixed crops located along an irrigation canal. A small herd of buffalo was found occasionally.

MR4 was located in the middle of Mae Rim district at an altitude of 348 m a.s.l. This area was defined as mixed deciduous forest according to a large-scale on the land use map. The fly traps were set in a small longan orchard and a small human community was found nearby.

MR5 was located in a big valley at an altitude of 746 m a.s.l. The fly traps were set in a lychee orchard near an irrigation stream.

MR6 was defined as a lowland village located in the south-eastern area of Mae Rim district at an altitude of 335 m a.s.l. This site was located close to Huay Tung Tao Lake which is a large man-made lake. The fly traps were placed in an open area along with the lake.

4. General description of study sites in Hang Dong district

Hang Dong district is a suburban area located to the west of Mueang Chiang Mai district. The co-ordinates, land use types, and elevation of the study sites in Hang Dong district are given in Table 3.

**Table 3.** The study sites in Hang Dong district.

| **Code** | **UTM Coordinate** | | **Location** | **Sub-district** | **Land use type** | **Altitude**  **(m a.s.l.)** |
| --- | --- | --- | --- | --- | --- | --- |
|  | **x** | **y** |  |  |  |  |
| HD1 | 483280 | 2077488 | Ban Pong | Bang Pong | Paddy field (PF) | 498 |
| HD2 | 486521 | 2074391 | Ban Pong | Ban Pong | Mixed deciduous forest (MDF) | 363 |
| HD3 | 491893 | 2071744 | Nhong Kwai | Nhong Kwai | Disturbed deciduous forest (DDF) | 332 |
| HD4 | 489894 | 2068367 | Namphrae | Namphrae | Mixed orchard (MO) | 326 |
| HD5 | 498572 | 2066598 | Nam Thong | Sob Mae Kha | Paddy field (PF) | 303 |
| HD6 | 492895 | 2065575 | Nhong Keaw | Nhong Keaw | Mixed orchard (MO) | 306 |


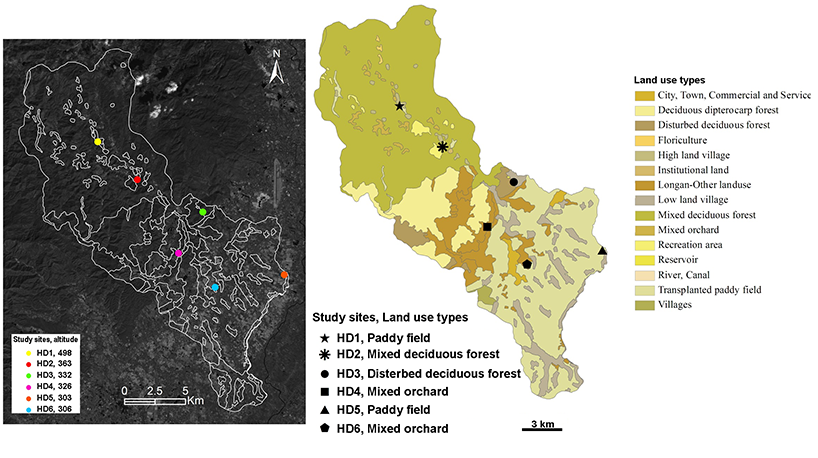


**Figure 3** Maps representing six study sites located in Hang Dong district.

HD1 was a mountainous area at an altitude of 498 m a.s.l. Adult flies were sampled in the valley, nearby the banana and sugar cane plantations area. A small human community comprising households, resorts, and a school was found around this area.

HD2 was located at an altitude of 363 m a.s.l. and surrounded by a human community, resorts, and restaurants. However, this area was classified as mixed deciduous forest according to the large-scale land use map. The traps were placed in an open area close to a small natural stream. A small cow herd was found occasionally around the study site.

HD3 was classified as disturbed deciduous forest at an altitude of 332 m a.s.l. The traps were set along the roadside close to a bee farm. A few houses and temples were located around this area.

HD4 was at an altitude of 356 m a.s.l. The fly traps were set in a public park situated among longan orchards.

HD5 was classified as a paddy field located at an altitude of 303 m a.s.l. Fly sampling was carried out in a small public park surrounded by a human community and close to a small dam of the Ping River.

HD6 was located in the Hang Dong municipal area at an altitude of 306 m a.s.l. This area represented a human community with rice plantations and mixed orchards. Fly sampling was conducted near a longan plantation because the land use of this site was defined as a mixed orchard. A few cows and horses were also were occasionally present in this area.

References

1. Ngoen-klan, R. Evaluation of Climatic and Physical Factors Influencing House Fly *Musca domestica* Linn and Blow Fly *Chrysomya megacephala* Fabricius Populations in the Urban and Suburban Areas of Chiang Mai, Thailand by Using Geographic Information System (GIS). PhD Thesis, Chiang Mai University, Chiang Mai, Thailand, 2011. Available online: http://repository.cmu.ac.th/handle/6653943832/29449 (accessed on 1 July 2018).
2. Rogers, D.J.; Williams, B.G. Monitoring trypanosomiasis in space and time. *Parasitology* **1993**, *106*, S77–S92.

© 2018 by the authors. Submitted for possible open access publication under the terms and conditions of the Creative Commons Attribution (CC BY) license (http://creativecommons.org/licenses/by/4.0/).
