# Supplementary material for: Spatial Distribution of Forensically Significant Blow Flies in Subfamily Luciliinae (Diptera: Calliphoridae), Chiang Mai Province, Northern Thailand: Observations and Modeling Using GIS
Source: Insects. 2018 Dec 3;9(4):181. doi: 10.3390/insects9040181 (PMC6315425; doi:10.3390/insects9040181)
Supplement: Supplementary file 1 [file insects-09-00181-s001.zip › Suppl/File S2 General description of Thailand climate.docx]

General Description of Thailand Climate during 2009–2010

According to the Thai Meteorological Department [1], the climate of Thailand can be divided into three seasons as follows:

(1) Summer or pre-monsoon season (mid-February to mid-May). This is the transitional period from the northeast to southwest monsoons. The weather becomes warmer, especially in the northern part of Thailand. April is the hottest month of summer.

(2) Rainy or southwest monsoon season (mid-May to mid-October). The southwest monsoon prevails over Thailand and abundant rain occurs over the country. The wettest period of the year is August to September. The exception is found at the east coast of southern Thailand, where abundant rain remains until the end of the year and November is the wettest month of the rainy season in Southern Thailand.

(3) Winter or northeast monsoon season (mid-October to mid-February). This is the cold period of the year especially in December and January in northern Thailand but there is a great amount of rainfall in the east coast of southern Thailand, especially during October to November.

As reported by the Thai Meteorological Department during 2009–2010, at the beginning of 2009, the weather was cold. Monthly rainfall was below average in summer. Hot weather started with a low pressure cell covering the northern part of Thailand from March to early May 2009. The beginning of the rainy season was characterized by increasing rainfall during the second week of May. A low pressure trough with the prevailing southwest monsoon resulted in rain across the country. Additionally, the tropical depression that weakened from the typhoon, Ketsana, moved through north-eastern Thailand on September 30 and caused torrential rain in Thailand with flash flooding in some areas at the end of September to the early of October 2009. Nevertheless, the total rainfall of Chiang Mai Province was below average except for October 2009 (Table 1), which was the wettest period of the year. The maximum rainfall was 223.4 mm in October 2009. The average temperature during the rainy season (June 2009–October 2009) was about 27.5–28.0 °C.

In late October to November 2009, a major high pressure system brought cool weather to the northern part of Thailand. The lowest temperature in winter was recorded in December 2009 (22.5°C). At the beginning of summer, the weather remained cold (February 2010), then the temperature increased and reached its peak in April 2010 (31.6°C).


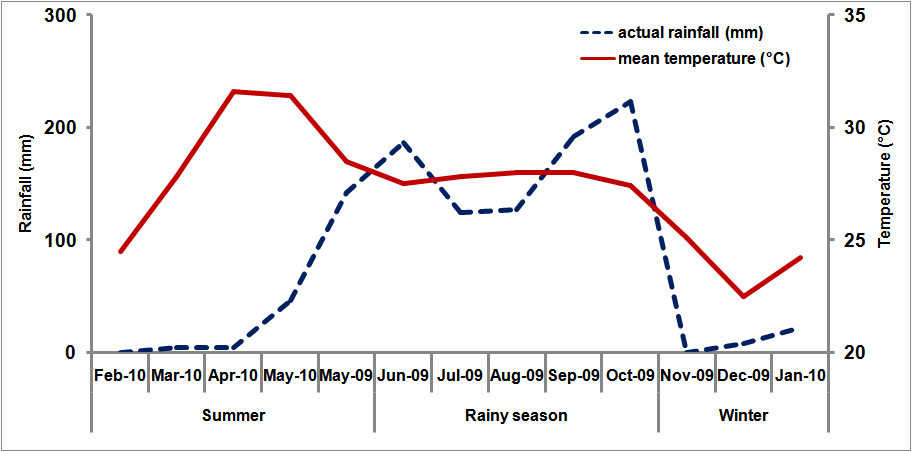


**Figure 1.** Monthly fluctuations in average temperature (°C) and actual rainfall (mm) recorded at weather station in Chiang Mai Province during May 2009–May 2010 (data from Thai Meteorological Department 2012).

**Table 1.** Monthly report on temperature (°C) and rainfall (mm) in Chiang Mai province during May 2009–May 2010 (Thai Meteorological Department 2010).

| **Season** | **Month** | **Temperature (°C)** | | **Rainfall (mm)** | |
| --- | --- | --- | --- | --- | --- |
|  |  | **Mean Temperature** | **Above/Below Average *** | **Actual Rainfall** | **Above/Below Average *** |
| Summer | 10 Feb. | 24.5 | 1.2 | 0 | −9.2 |
|  | 10 Mar. | 27.8 | 1.1 | 4.3 | -13 |
|  | 10 Apr. | 31.6 | 2.8 | 3.9 | −50.6 |
|  | 10 May | 31.4 | 3.3 | 46.4 | −109 |
|  | 9 May. | 28.5 | 0.4 | 142 | −13.4 |
| Rainy Season | 9 Jun. | 27.5 | 0 | 186.4 | 0 |
|  | 9 Jul. | 27.8 | 0.7 | 124 | −33.6 |
|  | 9 Aug. | 28 | 1.3 | 126.8 | −97.6 |
|  | 9 Sep. | 28 | 1.4 | 191.7 | −10.7 |
|  | 9 Oct. | 27.4 | 1.5 | 223.4 | 106.8 |
| Winter | 9 Nov. | 25.1 | 1.3 | 0 | −51.4 |
|  | 9 Dec. | 22.5 | 1.4 | 7.5 | −10.6 |
|  | 10 Jan. | 24.2 | 3.3 | 21.7 | 14 |

* Average data based on Thai Meteorological Department reports during 1981–2010.

Reference

1. Thai Meteorological Department. Thailand weather report 2009-2010. Available online: http://www.tmd.go.th (accessed on 29 October 2015).

© 2018 by the authors. Submitted for possible open access publication under the terms and conditions of the Creative Commons Attribution (CC BY) license (http://creativecommons.org/licenses/by/4.0/).
